# Embryonic Expression and Function of the *Xenopus* Ink4d Cyclin D-Dependent Kinase Inhibitor

**DOI:** 10.4172/2168-9296.1000133

**Published:** 2014-02-15

**Authors:** Joanne R. Doherty, Lisa M. Nilsson, Emin Kuliyev, Haiqing Zhu, Rose Matthew, John L. Cleveland, Paul E. Mead, Martine F. Roussel

**Affiliations:** 1 Department of Pathology, St. Jude Children’s Research Hospital, TN, USA; 2 Department of Biochemistry St. Jude Children’s Research Hospital, TN, USA; 3 Department of Tumor Cell Biology, St. Jude Children’s Research Hospital, TN, USA; 4 Department of Cancer Biology, The Scripps Research Institute, Scripps Florida, FL, USA; 5 Sahlgrenska Cancer Center, University of Gothenburg, Gothenburg, Sweden

**Keywords:** *Xenopuslaevis*, Cyclin-dependent kinase inhibitor, Ink4d, Cdkn2d, Cell cycle

## Abstract

Here we report the cloning and functional characterization of the cyclin D-dependent kinase 4 and 6 (Cdk4/6) inhibitory protein Cdkn2d/p19^Ink4d^ of *Xenopuslaevis* (*Xl*-Ink4d). *Xl*-*Ink4d* is the only *Ink4* family gene highly expressed during *Xenopus* development and its transcripts were detected maternally and during neurulation. The *Xl*-Ink4d protein has 63% identity to mouse and human Cdkn2d/p19^Ink4d^ and its function as a negative regulator of cell cycle traverse is evolutionary conserved. Indeed, *Xl*-lnk4d can functionally substitute for mouse Cdkn2d in binding to mouse Cdk4 and inhibiting cyclin-D1-dependent CDK4 kinase activity. Further, enforced expression of *Xl*-lnk4d arrests mouse fibroblasts in the G1 phase of the cell cycle. These findings indicate that CDKN2d/p19^Ink4d^ is conserved through vertebrate evolution and suggest *Xl*-lnk4d may contribute to the development of *Xenopuslaevis*.

## Introduction

Regulation of the cell cycle is intimately involved in development, where cell cycle arrest is coordinately controlled with terminal differentiation. Progression through the cell cycle is driven by cyclin-dependent kinases (Cdks) and their obligate binding partners, the cyclins. Cyclin/Cdk complexes are regulated by two families of Cdk inhibitory proteins (CKIs), the Cip/Kip family (Cdkn1) and the Inhibitors of Cdk4 (Ink4) family (Cdkn2) [1]. Enforced expression of CKIs induces cell cycle arrest by binding to and inhibiting cyclin-dependent Cdk activity. Specifically, Cip/Kip family members negatively regulate cyclin E-Cdk2, cyclin A-Cdk2, and cyclin B-Cdk1 complexes, whereas Ink4 proteins bind to and inhibit Cdk4 and Cdk6 kinases, by preventing their binding to D-type cyclins [1]. In turn, the retinoblastoma tumor suppressor protein Rb cannot be phosphorylated, and this prevents the release of E2F transcription factors that regulate the expression of genes that are necessary for entry and progression through the DNA synthetic (S) phase of the cell cycle [2].

Orthologues of *Ink4* genes are conserved throughout vertebrate evolution. *Fugu rubripes*, the most evolutionary distant organism analyzed, harbors two *Ink4* genes; *CDKN2b*/*Ink4b* and *CDKN2d*/*Ink4d* [3]. In the amphibian species *Xenopustropicalis,* three *Ink4* genes have been annotated in the draft genome sequence, *CDKN2b*/*Ink4b*, *CDKN2c*/*Ink4c* and *CDKN2d*/*Ink4d* (Joint Genome Institute, genome. jgi-psf.organd www.metazome.net). These three *Ink4* orthologues are likely conserved in *X. laevis* because of the high degree of similarity between the two *Xenopus* species. In mammals there are four *Ink4* genes, *CDKN2a*/p16^Ink4a^, *CDKN2b*/p15^Ink4b^, *CDKN2c*/p18^Ink4c^ and *CDKN2d*/p19^Ink4d^ [4–7].

In mice, Cdkn2d/p19^Ink4d^ and Cdkn2c/p18^Ink4c^ are the only two Ink4 family members expressed during embryonic development, where p19^Ink4d^ is first detected at embryonic day (E) 11.5 and where p18^Ink4c^ expression initiates at E13.5 [8,9]. Targeted deletion of either or both genes does not disrupt mouse development; thus, alone neither is essential for embryogenesis [10–12]. However, p19^Ink4d^ and p18^Ink4c^ are required to regulate cell cycle arrest and quiescence in specific cell contexts. For example, loss of *Ink4c* and *Ink4d* in the mouse induces male sterility due to a block in meiosis-I [12]. In the Central Nervous System (CNS) *Ink4d*-null mice progressively lose hearing due to cell cycle re-entry of sensory hair cells within the organ of Corti, followed by apoptosis [13]. Moreover, p19^Ink4d^ is required for proper mouse tooth development [14,15]. Further, *Ink4d* and *Cdkn1b*/*p27^Kip1^* together are necessary to maintain cerebral cortex neurons and retinal progenitor cells in a post-mitotic state, and for postnatal survival [16,17]. In the mouse, p18^Ink4c^ is induced during myogenic differentiation [18], is transiently expressed in granule neuron progenitors to time their exit from the cell cycle [19], and is required to maintain the hematopoietic stem cell progenitor pool [20]. Thus, in mice both p19^Ink4d^ and p18^Ink4c^ contribute to the induction and/or maintenance of a post-mitotic state in differentiated tissues.

Here we evaluated the expression and function of *Ink4* genes in early *Xenopuslaevis* development. Only one *Ink4* gene is highly expressed during *Xenopus* development and this encodes a protein, *Xl-Ink4d*, that was highly similar to the mouse orthologuep 19^Ink4d^, where *Xl-Ink4d* can bind to and inhibit mouse Cdk4 kinase activity on Rb, and is sufficient to provoke G1 arrest in mouse fibroblasts.

## Materials and Methods

### Cloning of *Xenopus Ink4* genes

A 139 base pair fragment was amplified from *Xenopuslaevis* genomic DNA by PCR using published degenerate oligonucleotide primers predicted to amplify the first 139 bp region of exon 2 of *Ink4-*like genes [3]. This fragment was used as a probe to screen a *Xenopuslaevis* adult spleen library (Lamda Zap Express) using standard methods [21]. Several clones were obtained and sequenced; however, only a single *Ink4*c DNA sequence was found (noted *Xl-Ink4d1*) that was similar to mouse and human *Cdkn2d/p19^Ink4d^*. Sequence analysis was performed using the DNA star software package (Lazergene).

### Production of *GST-Xl-Ink4d* and *GST-Mm-Ink4d* fusion proteins and *Cdk4* binding assays

The *Xl*-*Ink4d1* and mouse *Ink4d*(*Mm-Ink4d*) coding sequences were cloned in frame with an *N*-terminal GST-tag into pGEX-5X-1 and pGEX-2T, respectively (Amersham). The pGEX plasmids were transformed into BL21-D bacteria and their expression was induced with Isopropyl β-D-Thiogalactoside (IPTG) (0.1 mM) for 2 hour according to the manufacturer’s instructions. GST-tagged proteins were purified using glutathione-sepharose (Amersham) according to the manufacturer’s instructions. The coding sequence of *Xenopuslaevis Cdk4* (*Xl*-Cdk4) was cloned into pCMVTNT^™^ (Promega) and transcribed and translated in the presence of [^35^S]-methionine using TNT^®^ Coupled Reticulocyte Lysate Systems according to the manufacturer’s instructions (Promega).

GST pull down assays were performed as described [22]. Briefly, *in vitro* transcribed and translated *Xl-Cdk4* (20 μl) was incubated with 1 μg of purified GST, GST-*Xl-Ink4d1* (*Xenopus*), or GST-*Mm*-*Ink4d* (mouse) proteins immobilized on glutathione sepharose. The mixture was incubated at 4°C for 2 hour and washed several times in IP kinase buffer (50 mM HEPES pH 7.5, 10 mM MgCl_2_, 1 mM DTT, 2.5 mM EGTA, 10 mM β-glycerophosphate, 0.1 mM sodium orthovanadate, 1 mMNaF). Bound proteins were denatured and separated on a 12% (w/v) polyacrylamide-SDS gel and visualized by autoradiography [23].

### *In vitro* kinase assays

*In vitro* Cdk4 kinase assays were performed as described [22,24], with minor modifications. Briefly, *Spodoptera frugiperda* Sf9 cells were infected with baculo viruses encoding mouse Cdk4 and cyclin D1. Lysates from these cells were immuno-precipitated with Protein A-Sepharose pre-adsorbed to a Cdk4 antibody (C-22, Santa Cruz Biotechnology). After overnight incubation at 4°C, increasing amounts of GST-*Xl*-Ink4d1, GST-*Mm*-Ink4d, or GST proteins were added to the reactions and incubated for 2 hour at 4°C. Immuno-precipitations were washed in IP kinase buffer (50 mM HEPES pH 7.5, 10 mM MgCl_2_, 1 mM DTT, 2.5 mM EGTA, 10 mM β-glycerophosphate, 0.1 mM sodium orthovanadate, 1 mMNaF) and *in vitro* kinase assays were performed using GST-Rb as substrate and [γ-^32^P]-ATP. The reactions were resolved by electrophoresis on 12.5% (w/v) poly-acrylamide-SDS gels, and analyzed by autoradiography [23].

### Virus infection and cell cycle analysis

Retroviruses were generated as described [25], including control MSCV-IRES-*GFP* virus as well as a MSCV-*Xl-Ink4d1-*IRES-*GFP* and MSCV-*Mm-Ink4d*-IRES-GFP viruses. NIH-3T3 mouse fibroblast cells were infected with retroviruses and cultured for 24 hour. Cells were trypsinized, permeabilized and stained with propidium iodide to stain DNA. The DNA content of GFP-positive cells was measured by fluorescence-activated cell sorting (FAC scaliber) and the data were analyzed using Cell quest software (Becton Dickinson).

### *Xenopus* embryo manipulations

*Xenopuslaevis* embryos were obtained, fertilized and microinjected as described [26]. Briefly, female frogs were induced to lay eggs by gonadotropin injection, fertilized in vitro with macerated testis and de-jellied with 3% (w/v) cysteine hydrochloride. Embryos were staged according to the *Xenopuslaevis* normal tables of development[27].

### Reverse transcription PCR and quantitative RT-PCR

Total RNA was isolated from *Xenopus* embryos at stages 2–41 using Qiashredder and Qiaeasy RNA isolation kit (Qiagen). RNA was reverse transcribed with Superscript II polymerase primed with oligod T and PCR amplified with Hot Star Taq DNA polymerase (Qiagen). For RT-PCR trace [α^32^P]-dCTP was included in the reaction to allow detection of the PCR product by autoradiography. Reactions were separated on pre-cast 10% (w/v) polyacrylamide Tris–Borate–EDTA gels (Bio-Rad), fixed, dried and exposed to X-ray film. For relative quantitative RT-PCR, reactions were performed on an Cycler thermocycler using iQ SYBR Green Supermix (Bio-Rad) and primers for *Ink4d1* and *ODC* (as an internal control). ODC C_T_ values were subtracted from *Ink4d1* C_T_ values (ΔC_T_) to normalize for input cDNA. Relative RNA levels were calculated by subtracting the ΔC_T stage40_ from ΔC_T_ (ΔΔCT) and using the calculation 2^−ΔΔCT^. Primers used to amplify *Xenopus Ink4d1*(forward; 5′-TTGTAGGGATGCACGGAATC-3′, reverse; 5′-ATGAACCGAATCCTTTGCAC-3′), *Ink4d*-Q (quantitative) (forward; 5′-TCCTGTCATTACCTTCCTTGCCCT-3′, reverse; 5′-TGGACAAGGTTGGGTGTTTCCTCT-3′), *Cyclin-D1* (forward; 5′-ATCTGGACAGGAACCTCATCACG-3′, reverse; 5′-GGACTCAATCTGTTCTTGGCACG-3′) [28], *CDK4* (forward; 5′-CACTGTGACCG ACGAAAGAT-3′, reverse; 5′-TTCCGTGGATCCCTAGTGG-3′), and *ODC*(forward; 5′-GTCAA TGATGGAGTGTATGGATC-3′, reverse; 5′-CCATTCCGCTCTCCTGAGCAC-3′).

### Immunoblotting

Embryos were lysed in ice-cold lysis buffer (120 mM NaCl, 50 mM Tris-HCl [pH 8.0], 0.5% NP-40, 1 mM EDTA, and Complete protease inhibitors [Roche]). Lysates were cleared of lipid and yolk by Freon extraction (http://spot.colorado.edu/~klym/) and protein concentration was determined using a BCA Protein Assay Kit (Pierce). Equal amount of protein was resolved on 15% (w/v) polyacrylamide-SDS gels and transferred onto nitrocellulose membranes. To detect *Xl*-Ink4d protein, we raised a rabbit polyclonal antibody to the *C*-terminal peptide of *Xl*-Ink4d1 (amino acid sequence: SQLAAILDPRLASIFELST) and affinity-purified the antibody using the same peptide. This peptide is unique to the predicted *Xenopus Xl*-Ink4d protein and its sequence is shared between *Xl*-Ink4d1, *Xl*-Ink4d2 and the Ink4d of X-Tropical is but not those of *Fugu*, Mouse or Human. Thus the antibody does not cross react with mouse p19^Ink4d^ protein (negative data not shown). Membranes were probed with an antibody against α-tubulin (Sigma) as a control for protein loading. Goat anti-rabbit HRP-conjugated secondary antibodies (Amersham) and Super Signal Dura (Pierce) were used to develop the blots.

### *In situ* hybridization of whole embryos

*In situ* hybridization was performed as described [26]. Anti-sense probes were generated as described by Kelley et al. [29]. Briefly, anti-sense probes were synthesized with digoxigenin-coupled UTP (Roche) and detected with alkaline phosphatase coupled to anti-digoxigenin Fab fragments (Roche) followed by the chromogenic reaction with NBT/BCIP (Vectstain).

## Results and Discussion

**Figure 1:**
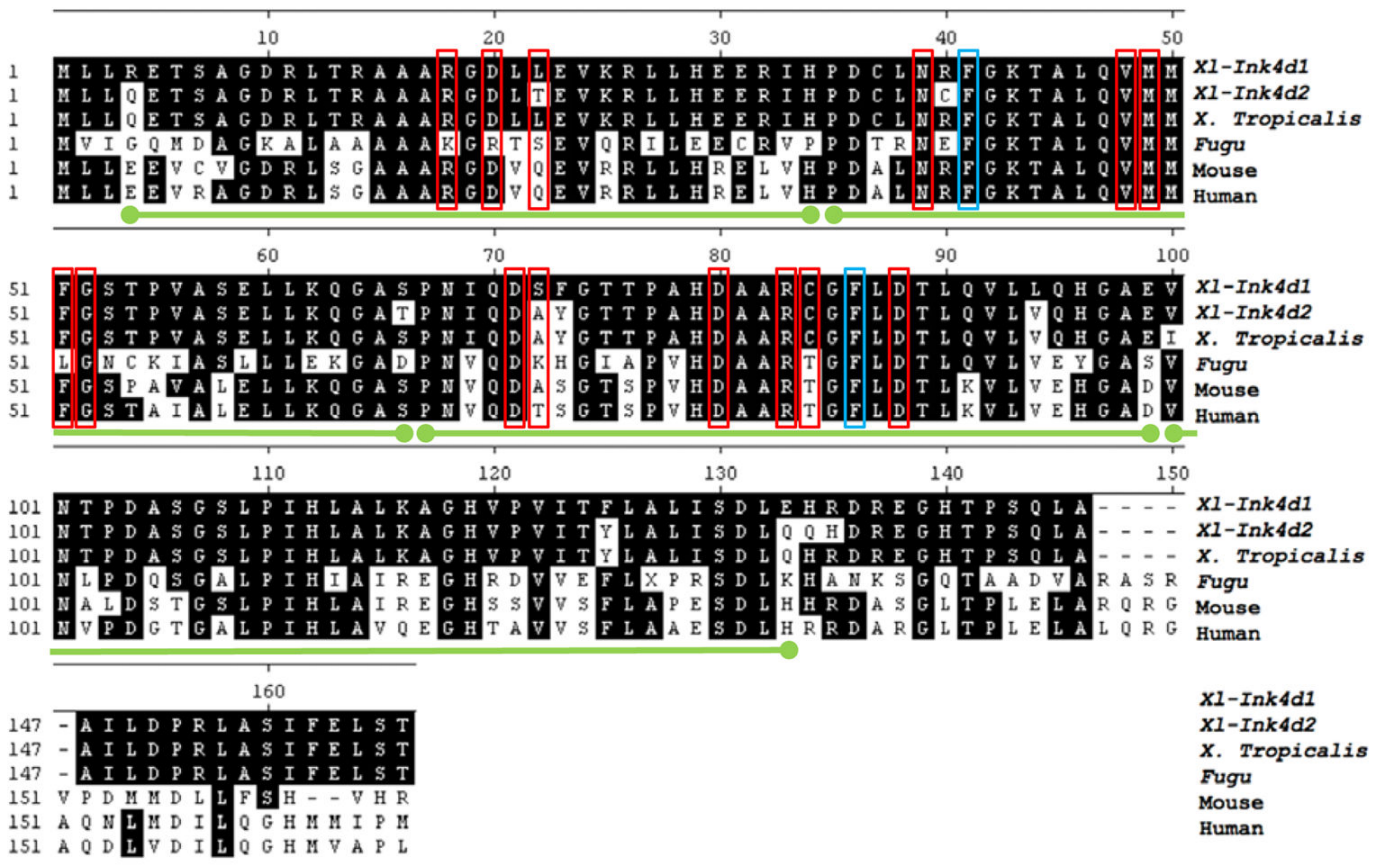
Predicted amino acid sequence alignments of Ink4d proteins. The predicted amino acid sequences of Ink4d proteins from *Xenopus tropicalis*, Fugu (*Fugu rubrides*), mouse (*Mus musculus*) and human *(Homo sapiens)* were compared to the two Ink4d proteins of *Xenopus laevis*. The *Xl-Ink4d1* cDNA was isolated from a library made from *Xenopus laevis* adult spleen, whereas *Xl-Ink4d2* was identified by blasting the NCBI database for similar expressed sequences in *Xenopus laevis* and represents a second allele of Ink4d. *Xl-Ink4d1* protein shares 63% amino acid identity with both the human and mouse Ink4d protein. The predicted four ankyrin repeats (green lines) as well as many of the amino acids that make contacts with CDK6 are conserved between mammals and *Xenopus* (red box = hydrogen bond interaction, blue box = non-polar interaction).

We identified a cDNA, *Xl*-*Ink4d1* that was most similar to mouse p19^Ink4d^ *(Mm-Ink4d)*. Blast search of the NCBI EST database using the *Xl*-*Ink4d1* sequence identified a second allele (*Xl*-*Ink4d2*) and the predicted amino acid sequences of these proteins was compared to the Ink4d proteins from other species (Figure 1). Alignment of the Ink4d proteins from *Homo sapiens* (human), *Musmusculus* (mouse), *Xenopustropicalis*, and *Fugurubripes* showed a high degree of amino acid conservation (63% amino acid identity between mouse and *Xenopus* Ink4d), suggesting a conserved function for this gene throughout evolution.

**Figure 2:**
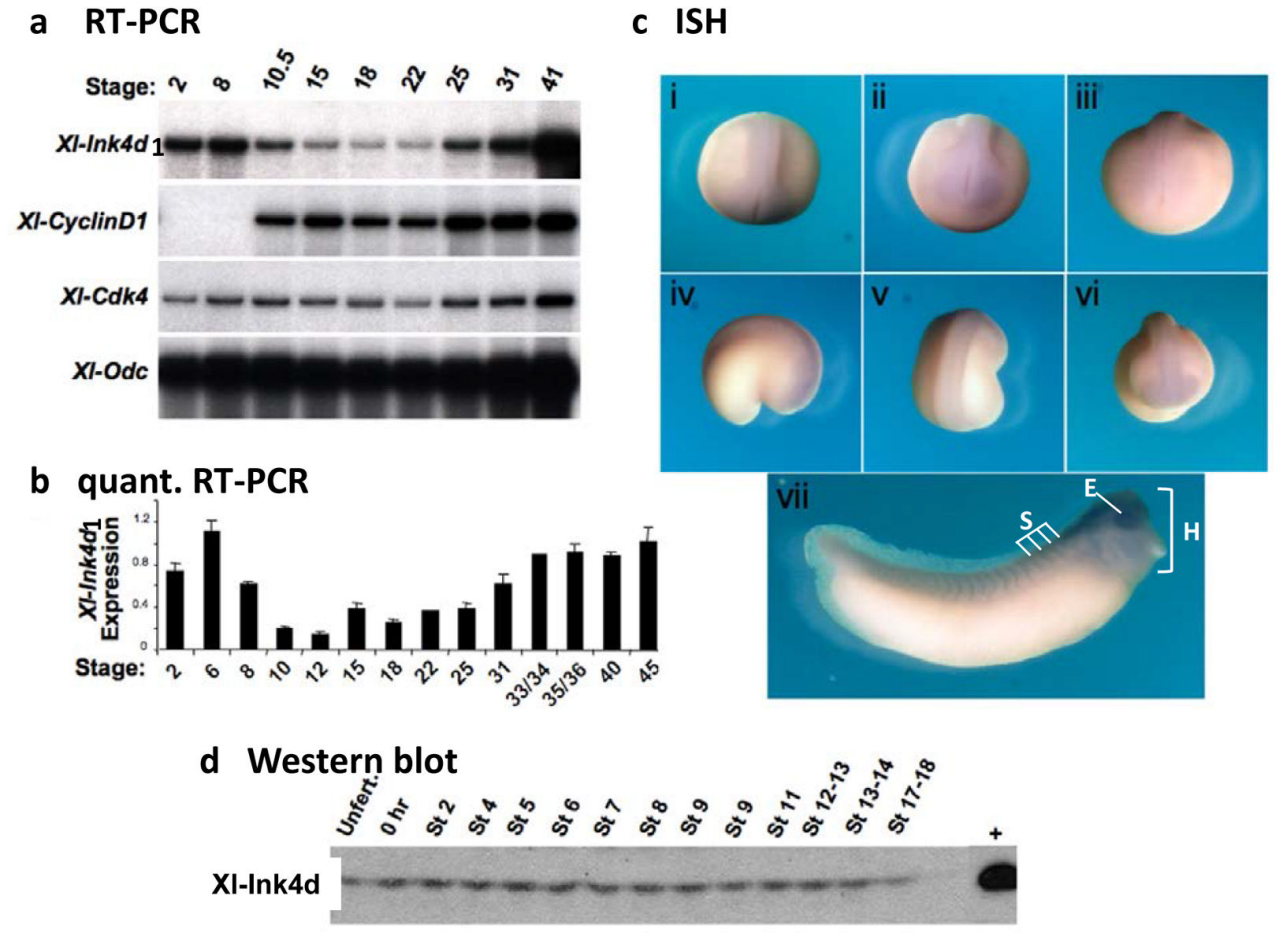
Expression of *Xl*-Ink4d during *Xenopus laevis* development. Embryos were staged according *Xenopus laevis* normal tables of development [27] and RNA was harvested at the indicated stages. (a) Reverse transcription PCR was performed on total RNA from staged embryos to amplify *Xl-Ink4d1*, *cyclin-D1*, *Cdk4* and *ODC* transcripts. Trace [α^32^P]-dCTP was added to the reactions and the PCR products were separated by gel electrophoresis. (b) Relative quantitative real-time PCR analysis of *Xl-Ink4d1* mRNA levels was performed using iQ SYBR Green Supermix (Bio-Rad), run and detected using an iCycler thermocycler (Bio-Rad). (c) In situ hybridization (ISH) for *Xl-Ink4d1* expression during early embryonic development. *Xl-Ink4d1* expression is detected at low levels in the dorsal anterior region of the developing tadpole. Stage 19 (i–iii); (i) dorsal view, (ii) anterior view, (iii) posterior view. Stage 22 (iv–vi); (iv) lateral view, (v) dorsal view, (vi) anterior view. Stage 32 (vii), trunk somites (S), eye (E) and head (H). (d) Immunoblotting of *Xl-*Ink4d from whole embryo lysates at the indicated stages using a polyclonal antibody directed against the *C*-terminus of the protein. Embryos microinjected with synthesized *Xl-Ink4d1* mRNA were harvested and run as a positive control (+).

Two *Ink4* genes are expressed during mouse embryogenesis, *Ink4c* and *Ink4d*, whereas *Ink4a* and *Ink4b* are only expressed in adult and aging animals [8,9]. As three *Ink4* genes were identified in *Xenopustropicalis*, we evaluated their expression by RT-PCR and real time PCR in developmentally staged *Xenopuslaevis* embryos (Figure 2a and 2b). *Xl*-*Ink4d1* transcripts were readily detected by RT-PCR and real time PCR indicating high-levels of this transcript during early development (Figure 2a and 2b). *Xl*-*Ink4b* transcripts were detected only by real-time PCR and their detection required five additional amplification cycles compared to that of *Xl*-*Ink4d1* mRNA; thus, significantly lower levels of *Xl-Ink4bvs. Xl*-*Ink4d1* are expressed during early *Xenopuslaevis* development. Moreover, *Xl-Ink4c* was undetectable by RT-PCR at any stage of embryonic development (not shown). This finding is in accord with the lack of*Xl-Ink4c* transcripts in publically available *Xenopus tropicalis* embryonic tissue EST libraries (Unigene (www.ncbi.nih.gov/Unigene) and in the Gurdon Institute *Xenopus tropicalis* EST database (http://informatics.gurdon.com.ad.uk)). Collectively, these data indicate that *Xl-Ink4d1* is the predominant Ink4 gene expressed during *Xenopus* development.

The *Xl*-*Ink4d1* transcript was maternally expressed in the egg and was evident before the mid-blastula transition (MBT, stage 8) when zygotic transcription begins. *Xl*-*Ink4d1* transcript levels decreased at the beginning of gastrulation (stage 10.5), remained low through neurulation (stage 22) and then increased at the end of neurulation (stage 25) (Figure 2a and 2b). *In situ* hybridization performed with *in vitro* transcribed antisense riboprobes revealed low levels of *Xl*-*Ink4d1* expression in the dorsal anterior region of the developing tadpole (Figure 2c). At neurula stages, *Xl*-*Ink4d1* was expressed in the neural plate (Figure 2c i–vi). By the late tail bud stage (stage 32a) *Xl*-*Ink4d1* expression was most prevalent in the brain, somite field, and eye and throughout the head (Figure 2c vii). *In situ* analysis using a probe that selectively detects the *Xl*-*Ink4d2* allele established that its expression was identical to that of *Xl*-*Ink4d1* indicating that expression of the two alleles is regulated in a similar manner (data not shown). Interestingly, mouse p19^Ink4d^ is expressed in the brain, spinal cord and dorsal root ganglia at embryonic day 13.5[9], suggesting a conserved function for *Ink4d* in neural development.

A rabbit polyclonal antibody generated against the *C*-terminus of *Xl*-Ink4d1, which is conserved between *Xl-Ink4d1* and *Xl-Ink4d2,* detected the *Xenopus* protein by immuno-blotting with high specificity (Figure 2d). Immunoblotting of whole embryo extracts from staged embryos demonstrated equal levels of *Xl*-Ink4d protein in the unfertilized egg and through early development (up to stage 18) indicating that although mRNA levels fluctuated during development the protein levels did not change.

**Figure 3:**
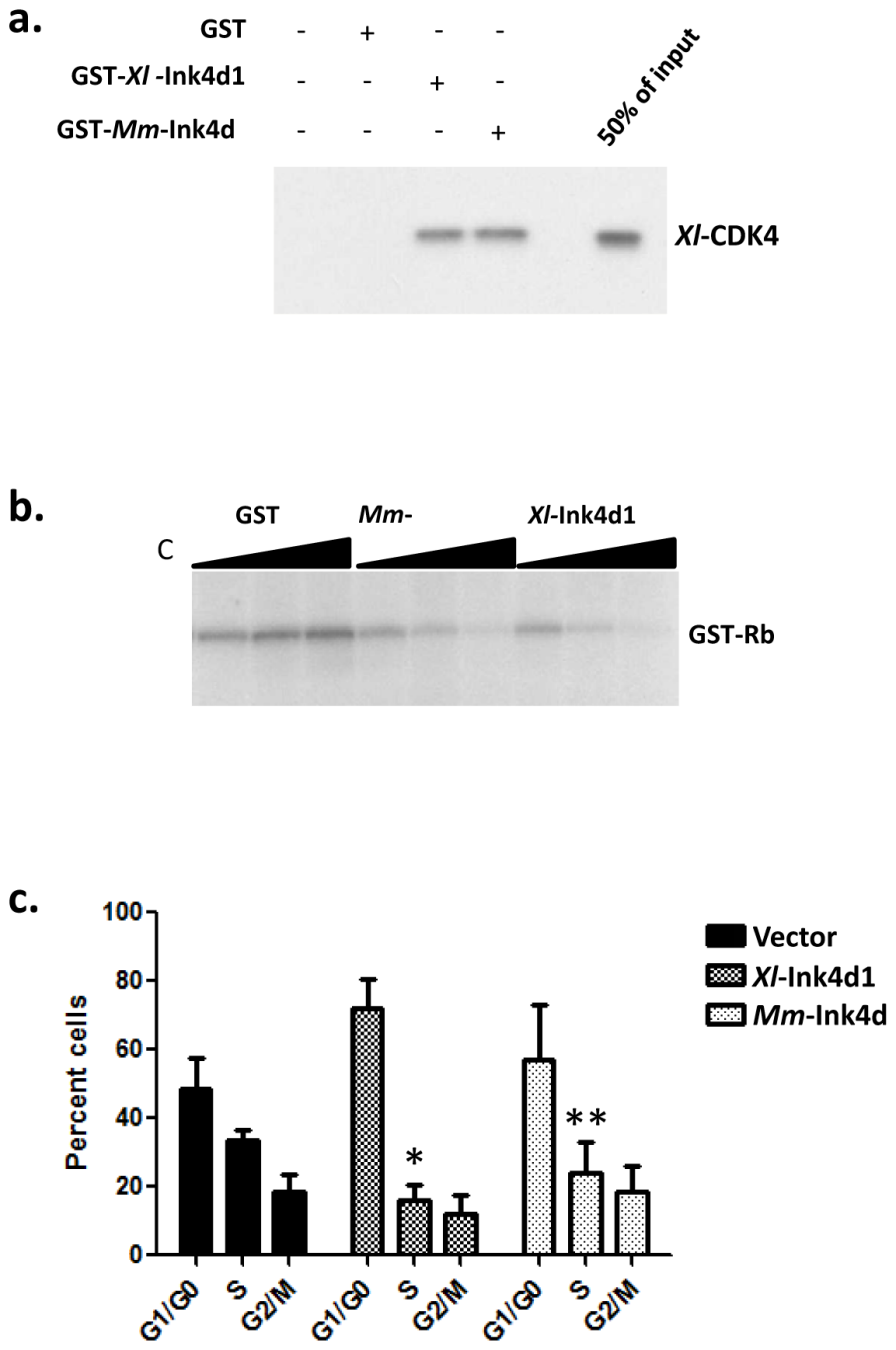
*Xl*-Ink4d is a cyclin D-dependent Cdk4 inhibitor. (a) The indicated GST fusion proteins were expressed in bacteria and purified on glutathione sepharose. The adsorbed sepharose was incubated with *in vitro* transcribed and translated [^35^S]-labeled *Xenopus* Cdk4 (*Xl*-Cdk4), washed and separated on an SDS-polyacrylamide gel. 50% of the input *Xl*-Cdk4 that was used for pull-downs was run to estimate the percent of starting material that bound to the GST fusion protein (50% input). (b) Cdk4 kinase reactions were run with increasing amounts of purified GST-*Xl*-Ink4d1 and GST-*Mm*-Ink4d (mouse Ink4d) fusion proteins. Active mouse Cdk4/cyclinD1 kinase complexes were generated from SF9 cells infected with baculoviruses encoding mouse Cdk4 and cyclin-D1. Kinase assays were performed with [γ-^32^P]-ATP using purified GST-Rb as substrate. (c) The cell cycle profile of NIH-3T3 mouse fibroblasts overexpressing vector-alone (black bars), *Xenopus Xl*-Ink4d1 (dark grey bars) or mouse *Mm*-Ink4d (light grey bars). NIH-3T3 cells were infected with retroviruses that co-express the indicated Ink4d genes and GFP, and the DNA content of GFP-positive cells was measured by propidium iodide staining and FACS analysis. The percentage of cells in G_1_/G_0_, S and G_2_/M phase is presented. Expression of either *Xl*-Ink4d1 (* p=0.006) or *Mm*-Ink4d (** p=0.05) shows a significant decrease in S phase compared to vector alone.

To determine if *Xl*-Ink4d functioned in a manner akin to that of mouse p19^Ink4d^, we tested its activity in a series of *in vitro* and *ex vivo* experiments. Mammalian *Ink4* proteins inhibit the kinase activity of Cdk4 by binding to Cdk4 and preventing its interactions with D-type cyclins [30]. We assessed the binding of *Xl-*Ink4d1, and of mouse Ink4d (*Mm-Ink4d*), to *Xenopuslaevis* Cdk4 (*Xl*-Cdk4) by GST-pull down experiments (Figure 3a). Both GST-*Xl*-Ink4d1 and GST-*Mm*-Ink4d fusion proteins bound to *Xl-*Cdk4, whereas, as expected, GST alone failed to interact with *Xl*-Cdk4 (Figure 3a).

Cdk4 phosphorylates and inactivates the function of the Rb tumor suppressor [1]. To test the ability of the *Xl-*Ink4d1 protein to inhibit Cdk4 kinase activity, mouse Cdk4-cyclin-D1 complexes were immuno-precipitated from Sf9 insect cell lysate that were co-infected with baculoviruses expressing mouse cyclin-D1 and Cdk4 [31], and these complexes were then incubated with increasing concentrations of the purified GST-Ink4d1 fusion proteins. Both *Xl*-Ink4d1 and *Mm*-Ink4d inhibited Rb phosphorylation in a dose-dependent fashion (Figure 3b). Therefore, *Xl*-Ink4d1 can inhibit the kinase activity of mouse cyclin-D1/Cdk4 complexes.

Cdk4 kinase activity drives cell cycle progression through the G1 phase of the cell cycle and inhibition of Cdk4 activity by overexpression of mouse Ink4d arrests cells in this phase [22]. To test if *Xl*-Ink4d1 could also arrest mouse fibroblasts in G1 phase we infected NIH-3T3 mouse fibroblasts with retroviruses encoding *Xl*-Ink4d1 or *Mm*-Ink4d under the control of the MSCV promoter together with GFP, which is expressed from the same transcript through an Internal Ribosomal Entry Site (IRES) present in the MSCV-IRES-GFP retroviral vector [32]. Infected cells were analyzed 32 hours after infection by Fluorescence Activated Cell Sorting (FACS) and the DNA content of GFP-positive cells was determined by propidium iodide staining. Overexpression of either *Xl*-Ink4d1 or *Mm*-Ink4d significantly reduced the percentage of cells in S phase compared to cells infected with control retrovirus (from 34% to 16% and 24%, respectively) and led to corresponding increases in the percentage of cells in G1 phase (from 49% to 72% and 57%, respectively), indicative of an accumulation of cells arrested in G1 (Figure 3c). Therefore, the *Xenopuslaevis Xl*-Ink4d1 protein can functionally substitute for the mouse *Mm*-Ink4d protein by binding to mouse Mm-Cdk4, inhibiting its cyclin D-dependent kinase activity and arresting mouse fibroblasts in G1 phase.

Altogether, our data demonstrate that *Xl-*Ink4d1 is a *bona fide Xenopus* orthologue of mammalian p19^Ink4d^ that is remarkably conserved throughout evolution. This is underscored by the facts that, similar to mouse Mm-Ink4d, *Xl-*Ink4d1 is expressed in neural regions during embryonic development and that *Xl-*Ink4d1 can substitute for mouse *Mm*-Ink4d in regulating the G1 phase of the cell cycle of mouse fibroblasts. The high levels of Ink4d expression during *Xenopuslaevis* development in neuronal tissues suggests that, akin to its mammalian homolog [16,17], *Xl-*Ink4d serves an important function in *Xenopus* embryonic brain development, and this deserves further examination.
